# The effect of blood feeding on insecticide resistance intensity and adult longevity in the major malaria vector *Anopheles funestus* (Diptera: Culicidae)

**DOI:** 10.1038/s41598-022-07798-w

**Published:** 2022-03-09

**Authors:** Shüné V. Oliver, Candice L. Lyons, Basil D. Brooke

**Affiliations:** 1 Present Address: Centre for Emerging Zoonotic and Parasitic Diseases, National Institute for Communicable Diseases of the National Health Laboratory Service, 1 Modderfontein Road, Sandringham, Johannesburg, 2192 South Africa; 2 Wits Research Institute for Malaria, School of Pathology, Faculty of Health Sciences, University of the Witwatersrand, Johannesburg, 2193 South Africa; 3 Department of Biological Sciences, University of Cape Town, Rondebosch, 7701 South Africa

**Keywords:** Animal physiology, Entomology, Zoology

## Abstract

Insecticide-based vector control is key to the reduction and elimination of malaria. Although insecticide resistance is common in malaria vector populations, the operational implications are often unclear. High intensity pyrethroid resistance in the major malaria vector *Anopheles funestus* has been linked to control failure in Southern Africa. The aim of this study was to assess linkages between mosquito age, blood feeding and the intensity of pyrethroid resistance in two *An. funestus* laboratory strains that originate from southern Mozambique, namely the moderately pyrethroid resistant FUMOZ and the highly resistant FUMOZ-R. Resistance tended to decline with age. This effect was significantly mitigated by blood feeding and was most apparent in cohorts that received multiple blood meals. In the absence of insecticide exposure, blood feeding tended to increase longevity of *An. funestus* females and, following insecticide exposure, enhanced their levels of deltamethrin resistance, even in older age groups. These effects were more marked in FUMOZ-R compared to FUMOZ. In terms of programmatic decision-making, these data suggest that it would be useful to assess the level and intensity of resistance in older female cohorts wherever possible, notwithstanding the standard protocols for resistance testing using age-standardised samples.

## Introduction

Vector control is key to the reduction and elimination of malaria. A limited number of *Anopheles* species transmit malaria, and their control is primarily insecticide-based, relying on the deployment of insecticides via indoor residual spraying (IRS), insecticide treated nets (ITNs) and larviciding as a component of larval source management^1–3^. Until very recently, the number of insecticides available for use in malaria control was contained within only four classes—the pyrethroids, organochlorines (DDT only), carbamates and organophosphates. These collectively target only two neuronal sites within insects i.e., the voltage-gated sodium ion channel and acetylcholinesterase. Additional compounds recently developed for use in malaria control are the neonicotinoid clothianidin and the oxidative phosphorylation uncoupler chlorfenapyr^4–7^. These, amongst others still in the development phase, were formulated for vector control because of the burgeoning incidence of insecticide resistance in *Anopheles* malaria vector populations.

Insecticide resistance in vector populations is almost ubiquitous across malaria-affected regions^8^, and resistance management is therefore a necessary policy in many vector control programmes^9, 10^. Although there are several reports of resistance undermining control efficacy^11–14^, the operational implications of resistance are often unclear^15^. This is because surveillance for insecticide resistance in vector populations is typically assessed first by measuring phenotypes using standardised response-to-exposure assays^16^. These assays are based on the use of diagnostic insecticide concentrations that in no way resemble those used on ITNs and to spray walls during IRS operations. To address this issue, the current guidelines include the baseline diagnostic dose test followed by a stepwise assessment of resistance intensity if detected. The diagnostic dose is usually calculated as twice the lowest concentration that induces at least 99% mortality in a normally insecticide susceptible population following exposure for a set period—in most instances one hour. Resistance is thus diagnosed if mortality < 98% 24 h after the one hour exposure, and intensity can subsequently be measured in the same manner using dosages at 5X and 10X the diagnostic concentration^16^. These data can be used to make assessments about the potential operational impact of resistance, especially where IRS is used for control^17, 18^.

The standardised assays are generally effective at diagnosing the occurrence (diagnostic dose) and potential threat of resistance (intensity assessments) and are recommended for use on age-controlled samples that adequately represent the vector population from which the test mosquitoes are drawn. In order to make resistance data comparable across regions and even species, it is currently recommended that adult female mosquitoes aged less than 5 days be used for insecticide bioassays, and that these females should not have acquired any blood meals. It is however reasonable to expect that age^19^ and blood-feeding status^20, 21^ can measurably affect resistance phenotypes, as has previously been demonstrated^22^, and it is also important to note that it is only older, blood-fed females that transmit malaria^23^.

High-intensity insecticide resistant phenotypes are generally produced by multiple mechanisms, at least one of which is a major-effect factor around which stabilising co-factors have been selected^24^. A documented example is pyrethroid resistance in southern African populations of the major African malaria vector species *Anopheles funestus*. This resistant phenotype is primarily mediated by monooxygenase detoxification (cytochrome P450s CYP6P9 and CYP6P13)^25–27^ that is facilitated by increased expression of glutathione-S-Transferases (GSTs) and thickened cuticles^28, 29^. These collectively produce a moderate-to-high intensity resistance phenotype that has previously been linked to operational control failure in South Africa and Mozambique^30, 31^. Available evidence further suggests that this phenotype does not incur fitness costs in affected *An. funestus* populations^32^.

*Anopheles funestus* is a highly anthropophilic species and may be amongst the first anophelines to adapt to human hosts^33^. Adult female anophelines tend to take multiple blood meals in a single gonotrophic cycle, making them especially efficient vectors of malaria^34, 35^ that often carry greater parasite loads than other vector species^36, 37^. Furthermore, *Plasmodium* infection increases blood-seeking behaviour and probing^38, 39^. In terms of understanding malaria epidemiology where *An. funestus* occurs, and controlling populations of this species, important characteristics to consider are blood feeding habits, adult longevity and insecticide resistance, and how these interact with each other.

Given that older, blood-fed females are the epidemiologically significant sector of a vector population, small changes in longevity can result in significant changes in malaria transmission^40^. Furthermore, the intensity of the pyrethroid resistance phenotype in *An. funestus* varies with age, and the insecticide susceptibility of *An. funestus*, like other mosquitoes, has been demonstrated to increase with age^41^, while blood feeding tends to reduce insecticide toxicity in exposed adult mosquitoes^22, 42^. A single blood feed^42, 43^ as well as multiple blood meals reduce age-related increases in insecticide susceptibility^44^ by reducing the amount of oxidative stress induced by insecticide exposure^45^.

Given these parameters, the aim of this study was to assess linkages between age, blood feeding and the intensity of pyrethroid resistance expression in *An. funestus,* especially in older cohorts that have passed through successive gonotrophic cycles.

## Materials and methods

### Mosquito strains

Two laboratory-reared strains of *An. funestus* were used in this study: FUMOZ, a strain colonised from Southern Mozambique in 2000, and FUMOZ-R, which was selected from FUMOZ for resistance to pyrethroids. These strains are housed in the Botha de Meillon insectary at 25 °C (±2 °C) and 80% (± 5%) humidity, with a 12:12 h day/night photoperiod including dawn and dusk transitions. Larvae were maintained on a diet of powdered Beano™ dog biscuits and yeast (3:1) as previously described^46^. FUMOZ has moderate pyrethroid resistance intensity, and FUMOZ-R high resistance intensity^18^. Resistance in these strains is primarily attributed to overexpression of CYP6P9, CYP6P13 and GSTe2^25, 28^, and thickened cuticles^29^ as previously described.

### The effect of blood feeding on subsequent susceptibility to insecticide exposure

Samples of FUMOZ and FUMOZ-R adult females drawn for insecticide bioassays were divided into cohorts representing 3 different nutritional regimens. The first cohort (control group) was maintained on 10% sucrose ad libitum for the duration of their lives, with no blood meals. The second cohort was offered a single blood meal either at the age of 3, 7, 11, 15, 18 or 21 days post emergence, also with ad libitum access to sucrose. The third cohort was offered blood meals at the ages of 3, 7, 11, 15, 18 and 21 days post emergence i.e. six consecutive blood meals, also with ad libitum access to sucrose. At each age point, a sub-sample of adult females from each cohort was removed for insecticide resistance bioassays. Throughout their lives, males and females in all cohorts were caged together and allowed to mate. Females in all cohorts were allowed to oviposit twice weekly for the duration of the experiment.

Those female samples scheduled for blood feeding were fed to repletion on a single human volunteer (S.V. Oliver). The females were allowed to feed only on this volunteer and no other individuals were involved in the feeding procedure. Ethical approval for the use of invertebrate organisms was waived by the University of the Witwatersrand, Faculty of Health Sciences ethics committee: (Waiver number: S Oliver 03-01-2018). This waiver specifies that S.V. Oliver is authorised to blood feed mosquitoes as an essential part of the mosquito husbandry required for the project. Informed consent was provided by S.V. Oliver.

Four hours after feeding, cohorts of blood-fed female mosquitoes were exposed to either 0.05% (1X), 0.25% (5X) or 0.5% (10x) deltamethrin^16^. Only fully fed females were used for the exposures. This time gap was chosen as a previous study showed that the greatest transcriptional variation, both in gene up- and down-regulation was observed 3 h post blood meal^47^ As such, four hours was chosen to allow time for transcript changes to take effect. It also allowed time for the fully fed mosquitoes to be collected, and the feeding status of the females to be fully ascertained. This four hour period has previously been demonstrated to be enough time to measure phenotypic changes in insecticide tolerance in *An. arabiensis*^44^ and *An. funestus*^42^. It should be noted that blood-induced increases in insecticide tolerance peak 24 h after a blood meal^21^, but this timeline was not experimentally feasible with the multiple blood feeding set-up. The mosquitoes were allowed to feed on the arm of the volunteer. The arm was presented on the top of the mesh cage and the mosquitoes were allowed to feed to repletion. The volunteer did not use any scented soap or fragrances on the skin before feeding. The volunteer did not consume caffeine for four hours prior to the blood feeding. Alcohol and nicotine were not variables as the volunteer consumes neither.

The exposures were performed using the standard WHO bioassay method with treated papers of the required concentrations purchased from the WHO supplier at Universiti Sains Malaysia^16^. Exposed mosquitoes were allowed ad libitum access to 10% sucrose, and mortality was scored 24 h post exposure. Controls included a sample of unexposed mosquitoes and a sample exposed to solvent-only treated paper. Data from assays were not used for analysis if control mortality exceeded 10%. Exposed mosquitoes were not returned to the experimental pool. For each of the exposures, 20–25 females were used per tube. Numbers per treatment are summarised in Supplementary Table 1. The experiments were conducted over a period of three months during which the samples were collected as three separate cohorts originating from three separate egg batches. Within each cohort, adult females were split into three groups i.e. unfed, single fed and multiple fed groups as described earlier. This was to ensure that any potential environmental variations would affect all treatment groups equally.

### The effect of blood feeding and insecticide exposure on subsequent adult female longevity

One hundred and fifty adult females from each of FUMOZ and FUMOZ-R were either provided with a blood meal at the age of 3 days or remained unfed. Each of these cohorts was then split into three groups: unexposed, 1X exposed (exposed to 0.05% deltamethrin using standard WHO bioassays) or 10X exposed (exposed to 0.5% deltamethrin using standard WHO bioassays). Thirty surviving females were removed from each group 24 h post exposure and placed in a cage. They were offered ad libitum access to 10% sucrose, but no further blood meals. Their respective longevities were monitored daily, with cadavers removed every day until all specimens were dead.

### Statistical analysis

To determine the relative impacts of age and multiple blood meals on insecticide resistance intensity and mortality, data from FUMOZ and FUMOZ-R treatments were compared using a generalised linear model, with a quasibinomial distribution in R (v. 3.1.2). A quasibinomial distribution was chosen to account for overdispersion^48^. Mortality was used as the response variable, while age, bloodmeal and insecticide intensity (strain), and appropriate interactions, were used as predictor variables. The model we fit was as follows: model1 = glm(y ~ Group + Age + Treatment + Age*Treatment*Group,family = quasibinomial) where y is a bound variable alive/dead. This test was performed separately for the cohorts exposed to the standard (1X), medium (5X) and high (10X) deltamethrin dosages to determine if any predisposed advantage as a result of blood-feeding on age was carried over at higher-dose exposures to insecticides, or if this advantage was lost. For each test, the most parsimonious model was chosen as the model that best represented our data as per Crawley (2007)^48^.

A Kaplan–Meier estimator was used to assess longevity, with a Log-rank test used as a measure of significance at 95% confidence.

### Ethical approval

This study was performed as per the ethics waiver from the University of the Witwatersrand to S Oliver: 03-01-2018.

## Results

### The effect of blood feeding on subsequent susceptibility to insecticide exposure

#### Bioassays at the standard (1X) 0.05% deltamethrin concentration

**Figure 1:**
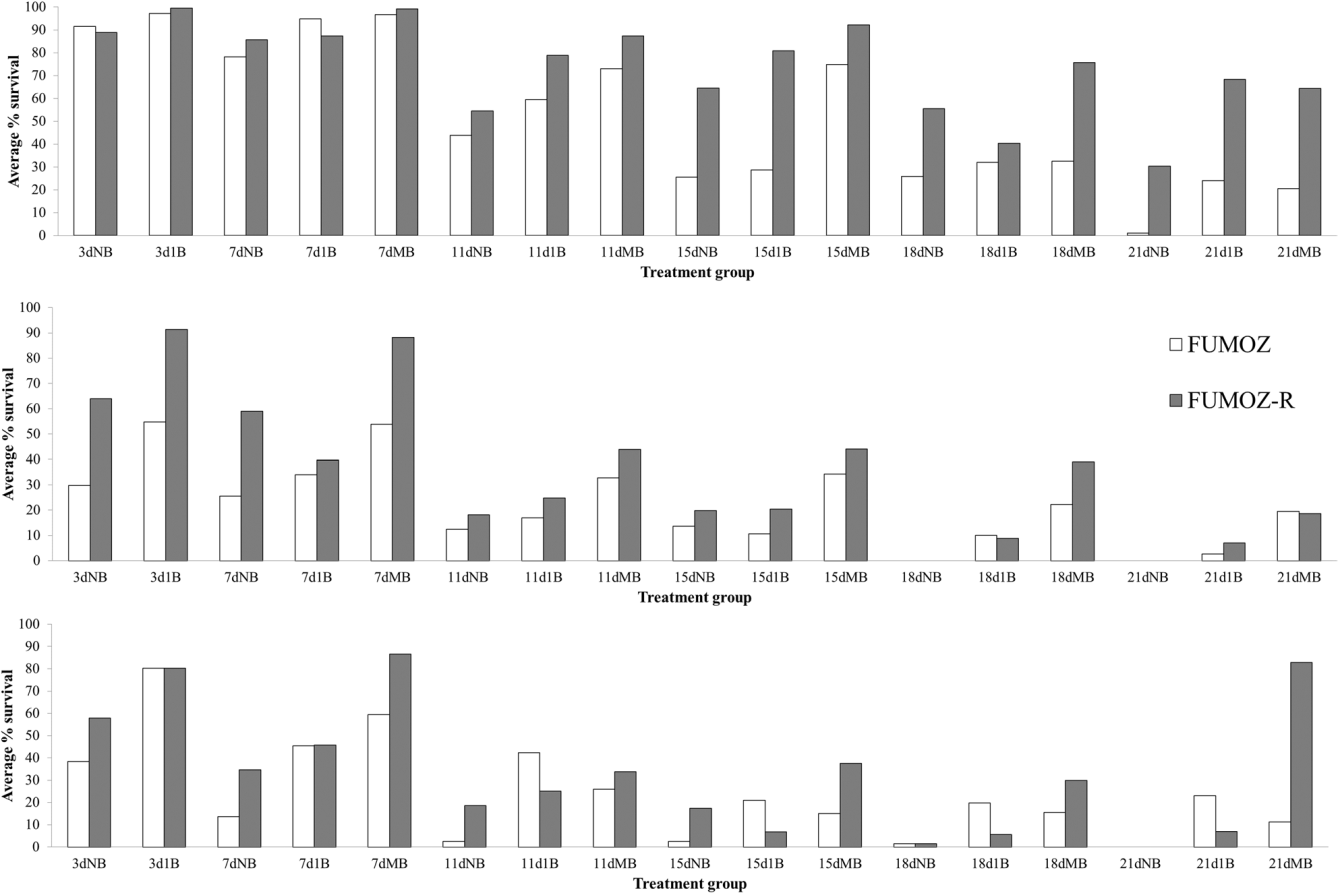
Survival of *Anopheles funestus* FUMOZ and FUMOZ-R laboratory-reared adult females after insecticide exposure bioassays by age and blood feeding status. Average survival (%) is given for FUMOZ (white) and FUMOZ-R (grey), grouped according to age and number of blood meals following exposure to (**a**) 1X Deltamethrin; (**b**) 5X Deltamethrin; and (**c**) 10X Deltamethrin. Treatment groups are as follows: ‘NB’ – no blood; ‘1B’ – one blood meal; ‘MB’ – multiple blood meals; while ‘3d’, ‘7d’, ‘11d’, ‘15d’, ‘18d’ and ‘21d’ refer to 3-day old, 7-day old, 11-day, 15-day, 18-day, and 21-day old treatment groups.

Survival was significantly decreased with increasing age in both strains. Multiple blood meals resulted in longer survival times for both strains, and there was no significant difference in survival between FUMOZ-R and FUMOZ. Females that were not fed blood experienced higher mortality than those fed blood. There was a significant two-way interaction between strain/colony and age (Group (FUMOZ)*Age), indicating that the effect of increased mortality with increased age differed between the two colonies (Table 1, Fig. 1).

**Table 1:** Results from generalised linear models with quasibinomial distribution of errors, showing the impact of insecticide resistance intensity (group), age (days), number of blood meals and the interaction between number of blood meals, age and group, on survival of *Anopheles funestus* at three different concentrations of deltamethrin (1X − 0.05%, 5X − 0.25% and 10X − 0.5%).

Concentration	Variable	Estimate	t-value	*P*-value
1 X	Intercept	3.34	11.58	***
	Group (FUMOZ)	0.29	0.67	0.5018
	Age	−0.15	−8.57	***
	Bloodmeals (multi)	0.54	3.11	**0.0021**
	Bloodmeals (No blood)	−0.7794	−4.46	***
	Group (FUMOZ)*Age	−0.09	−3.61	**0.0003**
5X	Intercept	1.49	6.87	***
	Group (FUMOZ)	−0.71	−6.41	***
	Age	−0.21	−10.25	***
	Bloodmeals (multi)	0.05	0.14	0.8853
	Bloodmeals (No blood)	−0.34	−1.09	0.2784
	Age*Bloodmeal (multi)	0.09	3.13	**0.0019**
	Age*Bloodmeal (No Blood)	−0.00	−0.05	0.9623
10X	Intercept	1.87	4.26	***
	Group (FUMOZ)	−0.71	−1.26	0.2082
	Age	−0.26	−6.32	***
	Bloodmeals (multi)	−2.36	−3.55	**0.0005**
	Bloodmeals (No blood)	−0.95	−1.75	0.0808
	Age*Bloodmeal (multi)	0.29	5.69	***
	Age*Bloodmeal (No Blood)	0.05	0.98	0.3276
	Group (FUMOZ)*Age	0.12	2.55	**0.0115**
	Group (FUMOZ)*Bloodmeal (multi)	2.49	2.71	**0.0072**
	Group (FUMOZ)*Bloodmeal (No Blood)	0.18	0.22	0.8296
	Group (FUMOZ)*Age*Bloodmeal (Multi)	−0.34	−4.83	***
	Group (FUMOZ)*Age*Bloodmeal (No Blood)	−0.22	−2.31	**0.0221**

All *p*-values with ‘***’ indicate p < 0.0001. Only the best models are shown per treatment, i.e. the non-significant interaction terms are removed from models to increase the model power.

Significant values are in bold.

#### Bioassays at the 5X 0.25% deltamethrin concentration

There was a significant difference in survival between the strains, with FUMOZ showing significantly higher mortality than FUMOZ-R. Increasing age resulted in a significant decrease in survival. There was no impact of blood meals on survival, and this was true whether the females had had multiple blood meals or no blood meals at all. There was, however, a significant interaction between age and blood meal, suggesting that multiple blood meals significantly improved survival of older females at this concentration (Table 1, Fig. 1). In most instances, the mortality of individuals within the resistant strain (FUMOZ-R) was lower as age increased, compared to mortality for the less resistant FUMOZ strain.

#### Bioassays at the 10X 0.5% deltamethrin concentration

There was no significant difference in survival between strains in general. There was, however, a significant difference by age, with older females experiencing increased mortality, when compared to their younger counterparts. The consumption of multiple bloodmeals also significantly impacted survival, with survival increasing with increased consumption of blood. There was no difference in mortality in the environmental control (unexposed) and solvent-only treated paper as there was no mortality in these treatments during the course of the 24-h period post-exposure.

There were significant two-way interactions between age and group/colony, indicating that older FUMOZ-R females experienced different mortality at this concentration when compared to older FUMOZ females i.e. older FUMOZ females died more readily than older FUMOZ-R females. The other significant two-way interaction was between colony and blood meal frequency, indicating that the influence of multiple blood meals on survival was expressed differently between colonies i.e. multiple blood fed FUMOZ-R females experienced higher survival rates by age cohort than FUMOZ females.

There were also two significant three-way interactions between Group*Age*Blood meal (multi or no blood). Although difficult to disentangle, data show that older resistant FUMOZ-R females, fed multiple blood meals, or even no blood meals, experienced reduced mortality at the 10X concentration when compared to FUMOZ females (Table 1, Fig. 1).

### The effect of blood feeding and insecticide exposure on subsequent adult female longevity

**Figure 2:**
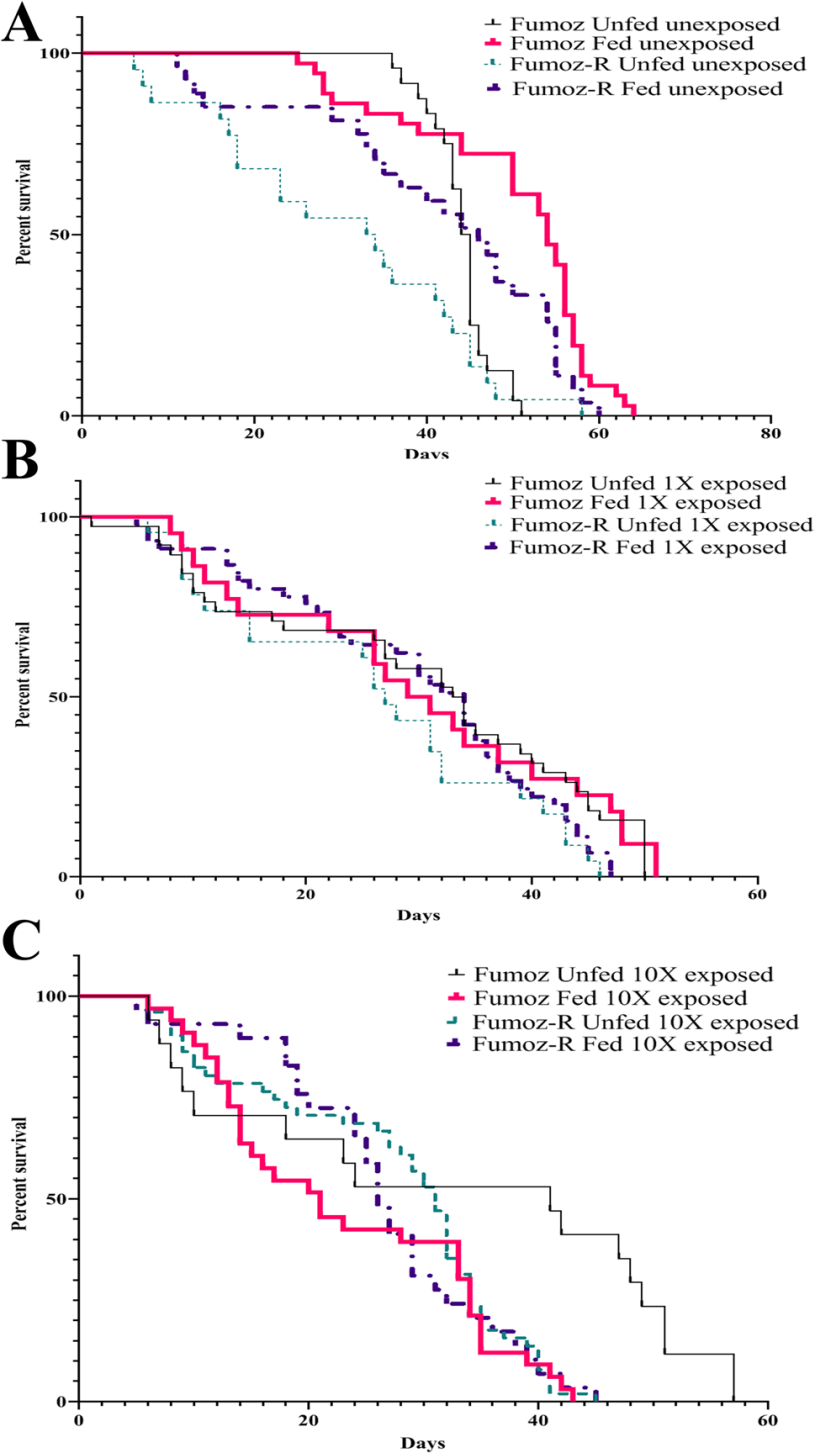
Longevity of *Anopheles funestus* FUMOZ and FUMOZ-R laboratory-reared adult females by deltamethrin exposure and blood feeding status. (**A**) Longevity in FUMOZ and FUMOZ-R unfed, unexposed adults. (**B**) Longevity in FUMOZ and FUMOZ-R after a single deltamethrin exposure in adults fed a single blood meal at age 3 days and exposed to a concentration of 0.05% (1X). (**C**) Longevity in FUMOZ and FUMOZ-R after a single deltamethrin exposure in adults fed a single blood meal at age 3 days and exposed to a concentration of 0.5% (10X).

Longevity was significantly greater in unexposed FUMOZ compared to FUMOZ-R, regardless of whether they were blood-fed (Log rank test: *p* = 0.02, χ^2^ = 5.32, DF = 1) or unfed (Log rank test: *p* = 0.04, χ^2^ = 4.34, DF = 1). Exposure to the standard concentration of deltamethrin (0.05%) tended to nullify this effect in both the blood-fed and unfed cohorts (Log rank test: unfed-*p* = 0.08, χ^2^ = 3.01, DF = 1; fed-*p* = 0.23, χ^2^ = 1.41, DF = 1), as did exposure to the 10X (0.5%) deltamethrin concentration (Log rank test: unfed-*p* = 0.21, χ^2^ = 1.54, DF = 1; fed-*p* = 0.49, χ^2^ = 0.46, DF = 1) (Fig. 2).

When examining the mean survival time to 50% mortality (St_(50)_), unfed and unexposed FUMOZ showed an St_(50)_ 3 days later than FUMOZ-R. When the unfed adults were exposed to 1X deltamethrin, both strains showed an St_(50)_ of 27 days. By contrast, when exposed to 10X deltamethrin, FUMOZ-R showed an St_(50)_ 11 days later than FUMOZ.

When fed, unexposed FUMOZ showed an St_(50)_ 9 days later than FUMOZ-R. When exposed to 1X deltamethrin the St_(50)_ of FUMOZ-R was 3 days later than that of FUMOZ. The effect was even more marked when exposed to 10X deltamethrin, where FUMOZ-R showed an St_(50)_ 9 days later than FUMOZ (Table 2).

**Table 2:** Average survival time (St_(50)_) of FUMOZ and FUMOZ-R after an initial bloodmeal and deltamethrin exposure.

Treatment	St_(50)_(days)		
	Fumoz	Fumoz R	Difference
0 blood unexposed	**45**	31	+ 14
1 blood unexposed	**55**	47	+ 8
0 blood exposed 1X	**34**	27	+ 7
1 blood exposed 1X	31	**34**	− 3
0 blood exposed 10X	**41**	31	+ 10
1 blood exposed 10X	21	**27**	− 6

Green treatment titles represent unfed cohorts and black treatment titles represent fed cohorts.

Significant values are in bold.

## Discussion

There is growing evidence of a direct association between insecticide resistance intensity and operational control effectiveness^11, 49, 50^. Although it is suggested that delayed mortality leads to sustained efficacy of long lasting insecticide treated nets (LLINs) in the presence of pyrethroid resistance^15^, it has recently been reported that populations in Burkina Faso show only minimal delayed mortality^51^. To fully understand the impact of resistance intensity on vector control operations, it is important to understand its’ effect on the survival of older, blood-fed females.

Insecticide resistance in the *An. funestus* strains used in this study is primarily mediated by metabolic detoxification^28, 41, 52^ although they differ in terms of resistance intensity^18^. In general, resistance tended to decline with age in both strains. This effect was significantly mitigated by blood feeding and was most apparent in FUMOZ-R. Mitigation of the loss of resistance with age was also most apparent in those cohorts that received multiple blood meals. Importantly, this mitigation effect was evident in those cohorts exposed to 5X and 10X the diagnostic dose, ultimately showing that blood feeding, and especially multiple blood feeding, tended to enhance the level and intensity of resistance to deltamethrin in both strains, and enabled the survival of a small proportion of older females even at the 10X dose.

A variety of factors can affect the longevity of adult mosquitoes. These include environmental factors such as humidity and temperature, as well as predation^53^. The larval environment and subsequent size of the adult also plays a role in longevity^54^. It has been demonstrated that insecticide resistant *An. gambiae* and *An. arabiensis* are likely to experience reduced longevity^44, 55^. Following this trend, the less resistant FUMOZ strain is evidently longer-lived than FUMOZ-R in the absence of exposure to insecticide. This difference however disappeared following exposure to deltamethrin at the 1X and 10X doses. In the absence of insecticide exposure, blood feeding tended to increase longevity, but this effect was lost after exposure to the diagnostic doses of insecticide. This observation was however made after an initial single blood meal, and not multiple blood meals. A comparison between the blood-fed cohorts shows that those not exposed to insecticide had greater longevity than those exposed to the 1× and 10X doses across both strains.

Although blood feeding did not necessarily increase longevity, especially following exposure to insecticide, it also did not cause any reductions in lifespan and, importantly, blood feeding bolstered the level and intensity of deltamethrin resistance in older female mosquitoes. This was most apparent in FUMOZ-R, suggesting a lower delayed mortality than FUMOZ. A recent study has independently demonstrated a reduction in delayed mortality in FUMOZ-R following repeated exposures to pyrethroid treated bed nets^56^.

It is suggested that this effect is related to oxidative stress. A previous study found that age-related changes in pyrethroid resistance were not associated with cytochrome P450 transcription, regardless of blood feeding status^41^. By contrast, FUMOZ-R has an increased capacity to withstand oxidative stress compared to insecticide susceptible *An. funestus*. This is mediated by high catalase and glutathione peroxidase activity^45^. In this scenario, multiple blood feeding reduces the oxidative burden in insecticide resistant mosquitoes, producing the downstream effect of reducing the oxidative burden induced by exposure to insecticide. This has previously been demonstrated by increased glutathione peroxidase and catalase activity in insecticide resistant *An. arabiensis* that had been provided multiple blood meals^45^. The significantly higher oxidative stress defence capacity of FUMOZ-R may underlie the ability of older females to survive deltamethrin exposure at the 10X dose.

Although this study was performed on laboratory strains, it has previously been shown that the insecticide resistance intensity of FUMOZ-R is comparable to that of wild Zambian *An. funestus*, and likely comparable to populations of this species in Mozambique from where the FUMOZ and FUMOZ-R strains are derived^18^. The high intensity resistance in these populations has been linked to reduced efficacy of pyrethroid impregnated bed nets under laboratory conditions^14, 18, 31, 57–61^.

The effect of continued exposure of mosquitoes to sub-lethal doses of insecticide is poorly understood, especially in the context of multiple blood meals. This study demonstrates that insecticide resistance intensity is amplified by multiple blood feeding. This highlights the importance of intensity screenings as part of routine surveillance because intensity assessments can be used to predict the operational significance of resistance in the field^16, 17^. The study also shows that the presence of high-intensity resistance has differential effects on the life history parameters of affected mosquitoes.

It is concluded that blood feeding tends to increase the longevity of *An. funestus* females and enhances their levels and intensity of pyrethroid resistance, even in older age groups. This is important in terms of measuring insecticide resistance in wild vector populations and making inferences about the possible impact of resistance on operational efficacy, especially because it cannot be assumed that older females will not be able to withstand the same levels of insecticide intoxication as younger females. In terms of programmatic decision-making, it should therefore be considered useful to assess the level and intensity of resistance in older female cohorts wherever possible, notwithstanding the standard protocols for resistance testing using age-standardised samples^16^.

## Supplementary Information

Supplementary Information.
