# Supplementary material for: The effect of blood feeding on insecticide resistance intensity and adult longevity in the major malaria vector Anopheles funestus (Diptera: Culicidae)
Source: Sci Rep. 2022 Mar 9;12:3877. doi: 10.1038/s41598-022-07798-w (PMC8907345; doi:10.1038/s41598-022-07798-w)
Supplement: Supplementary file 1 — Supplementary Information. [file 41598_2022_7798_MOESM1_ESM.docx]

Supplementary Table 1: Sample numbers of *Anopheles funestus* FUMOZ and FUMOZ-R laboratory-reared adult females used for insecticide susceptibility bioassays by concentration where 1X represents the standard WHO discriminating dose.

|  |  | FUMOZ | | | FUMOZ-R | | |
| --- | --- | --- | --- | --- | --- | --- | --- |
| Concentration | Age | No Blood | Single Blood | Multiblood | No Blood | Single Blood | Multiblood |
| 1X | 3 Day | 108 | 108 |  | 176 | 117 |  |
|  | 7 Day | 101 | 103 | 123 | 174 | 283 | 120 |
|  | 11 Day | 137 | 106 | 148 | 112 | 198 | 189 |
|  | 15 Day | 98 | 94 | 147 | 93 | 121 | 208 |
|  | 18 Day | 166 | 181 | 185 | 98 | 95 | 193 |
|  | 21 Day | 100 | 162 | 164 | 122 | 155 | 125 |
| 5X | 3 Day | 128 | 120 |  | 180 | 127 |  |
|  | 7 Day | 184 | 227 | 115 | 151 | 280 | 110 |
|  | 11 Day | 328 | 148 | 201 | 127 | 145 | 221 |
|  | 15 Day | 162 | 234 | 108 | 222 | 157 | 304 |
|  | 18 Day | 129 | 125 | 257 | 120 | 102 | 161 |
|  | 21 Day | 115 | 125 | 246 | 116 | 172 | 113 |
| 10X | 3 Day | 120 | 125 |  | 192 | 122 |  |
|  | 7 Day | 110 | 141 | 128 | 193 | 220 | 129 |
|  | 11 Day | 121 | 125 | 112 | 156 | 168 | 213 |
|  | 15 Day | 111 | 114 | 167 | 208 | 175 | 173 |
|  | 18 Day | 98 | 104 | 187 | 129 | 106 | 157 |
|  | 21 Day | 157 | 161 | 151 | 101 | 130 | 150 |
